# Dynamic Principal Component Analysis with Nonoverlapping Moving Window and Its Applications to Epileptic EEG Classification

**DOI:** 10.1155/2014/419308

**Published:** 2014-01-16

**Authors:** Shengkun Xie, Sridhar Krishnan

**Affiliations:** ^1^Department of Global Management Studies, Ted Rogers School of Management Studies, Ryerson University, 350 Victoria Street, Toronto, ON, Canada M5B 2K3; ^2^Department of Electrical and Computer Engineering, Ryerson University, 350 Victoria Street, Toronto, ON, Canada M5B 2K3

## Abstract

Classification of electroencephalography (EEG) is the most useful diagnostic and monitoring procedure for epilepsy study. A reliable algorithm that can be easily implemented is the key to this procedure. In this paper a novel signal feature extraction method based on dynamic principal component analysis and nonoverlapping moving window is proposed. Along with this new technique, two detection methods based on extracted sparse features are applied to deal with signal classification. The obtained results demonstrated that our proposed methodologies are able to differentiate EEGs from controls and interictal for epilepsy diagnosis and to separate EEGs from interictal and ictal for seizure detection. Our approach yields high classification accuracy for both single-channel short-term EEGs and multichannel long-term EEGs. The classification performance of the method is also compared with other state-of-the-art techniques on the same datasets and the effect of signal variability on the presented methods is also studied.

## 1. Introduction

Epilepsy is one of the most common chronic neurological disorders which affect about 50 million people worldwide [1]. The disorder is associated with abnormal brain neuronal activity which can be characterized by recurrent seizure [2]. Electroencephalography (EEG), as the most specific noninvasive method to define epileptogenic cortex, effectively reveals the characteristic findings in several epilepsy related syndromes [3]. As EEG signals are complex signals that are nonstationary, time-frequency domain methods based on wavelet transforms [4] have been proposed to locate the epileptic seizure pattern in both time and frequency domains. However, the development of reliable automated EEG-based tools for epilepsy diagnosis and seizure detection is still in its preliminary stage due to the lack of objective markers.

In this paper, we propose a new method, namely, dynamic principal component analysis (DPCA) with nonoverlapping moving window, to deal with both epileptic seizure detection and epilepsy diagnosis problems. DPCA [5] aims to extract important EEG signal features that explain major data variances, while nonoverlapping moving window is designed to potentially improve the classification performance for EEGs. Additionally, two feature extraction methods are proposed. The first one is based on the first few principal components, while the second one is to combine the first few principal components (PCs) with the signal energy measure in PC space. In epilepsy diagnosis, discriminative features are first extracted from normal and patient EEG signals using the DPCA method, followed by the mapping of each test EEG signal onto the constructed principal component subspace. Besides the validation of the proposed techniques for applying to EEGs, the effects of time variability, intersubject variability, and spatial variability of EEGs on the proposed methods are also tested.

Our main contributions in terms of the methodology development and the new application are as follows: (1) feature extraction via DPCA with non-overlapping moving window techniques to deal with univariate long-term signal; (2) applications of proposed methods in a novel way to biomedical signal classification problems with potential impact on computer-based epilepsy monitoring. The rest of the paper is organized as follows.  Section 2 describes the proposed detection schemes based on DPCA methodology.  Section 3 provides justifications of the application of the proposed method in epilepsy diagnosis and seizure detection by considering a set of short-term and long-term EEGs. Concluding remarks are provided in Section 4.

## 2. Methods

Epilepsy monitoring is usually a lengthy process to collect EEG signals at different stages of brain activities, including the stage of eyes open and eyes closed, the stage of interictal activity (i.e., the period between seizures), the stage of preictal, and the stage of ictal. Suppose that *y*_*i*_(*n*) with *n* = 1,2,…, *N* and *i* = 1,2,…, *g* are one-dimensional signals. Here, *n* and *i* are discrete time index and class index, respectively, with *N* being the total observational time and *g* being the total number of signal groups. The value of *N* for epilepsy monitoring is typically large; for example, *N* = 2^20^, and in the presented work *g* = 2 is defined to discriminate signals *y*_*i*_(*n*) as abnormal (i.e., in the presence of events of interest) and normal (i.e., at the absence of such events) classes. Suppose that *s*(*n*), where *n* = 1,2,…, N*, is a test signal from the same data source and our goal is to classify *s*(*n*) into either the normal group or the abnormal group by comparing the characteristics of data often obtained by some feature extraction methods. Typically, the test signal *s*(*n*) has a shorter time length than the training signal *y*_*i*_(*n*); that is, N* ≪ *N*. Our approach of signal discrimination is based on the classification of extracted features of a given test signal.

### 2.1. Experimental Datasets

**Figure 1:**
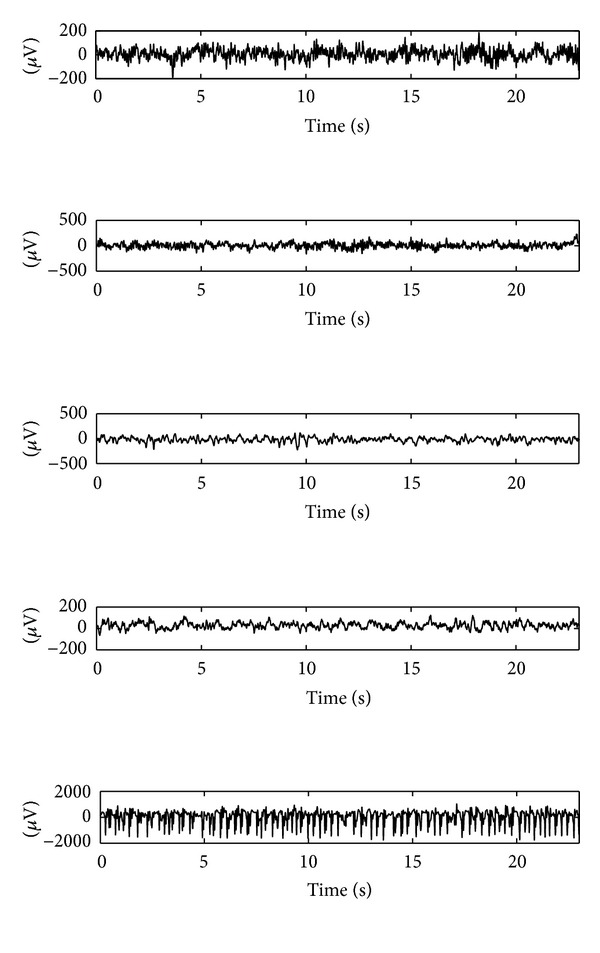
Examples of EEG segments from each of five sets (A, B, C, D, and E). From top to bottom: segment from A to segment from E.

**Figure 2:**
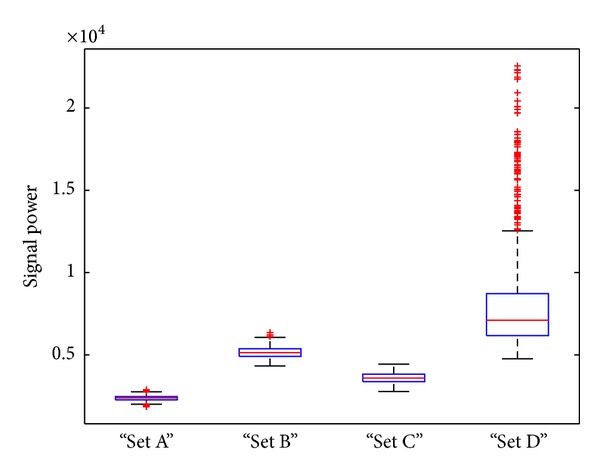
Box plots of average power of signal segments for datasets A, B, C, and D; the length of signal segments *l* = 512 is used.

The first EEG dataset (denoted by data set number 1) used in this work is a set of single-channel EEG data. This data is available online from the Epilepsy Center at the University of Bonn, Germany. Five sets of EEG signals have been included, which are denoted as Sets A, B, C, D, and E. Sets A and B consist of the EEG recordings taken from five healthy volunteers. Set A corresponds to the volunteers who were relaxed and in the awake state with eyes open; Set B is from the same volunteers with eyes closed; Sets C, D, and E were recorded from patients suffering from epilepsy with sensors at various spatial locations. Signals in Set C were recorded from the hippocampal formation of the opposite hemisphere of the brain to the epileptogenic zone and Set D was recorded from within the epileptogenic zone. Both Sets C and D contain only brain activity measured during seizure-free intervals, while Set E contains only seizure activity. Each group contains 100 single-channel scalp EEG segments of 23.6-second duration sampled at 173.61 Hz [14] and we refer to these signals as short-term signals. In order to accommodate the spatial and between objects variabilities of signals, we use Sets A, B, C, and D that contain both normal and abnormal signals for epilepsy diagnosis problem and use Sets A, B, C, D, and E for seizure detection. Examples of EEG segment are shown in Figure 1 and average signal powers calculated using all signals in the dataset for Sets A, B, C, and D are reported in Figure 2. One can see that the signal powers of some types of epileptic EEGs (D and E) are larger than the normal ones (A and B), in particular, for the seizure type of signal (i.e., E). This implies that signal energy may be an important feature for discrimination of normal and epileptic signals. However, the method purely based on signal powers cannot be used to discriminate signals of Set C (recorded from the nonepileptic zone) from normal signals; this may suggest that identification of spatial location of epileptic zone is required for epilepsy diagnosis.

**Figure 3:**
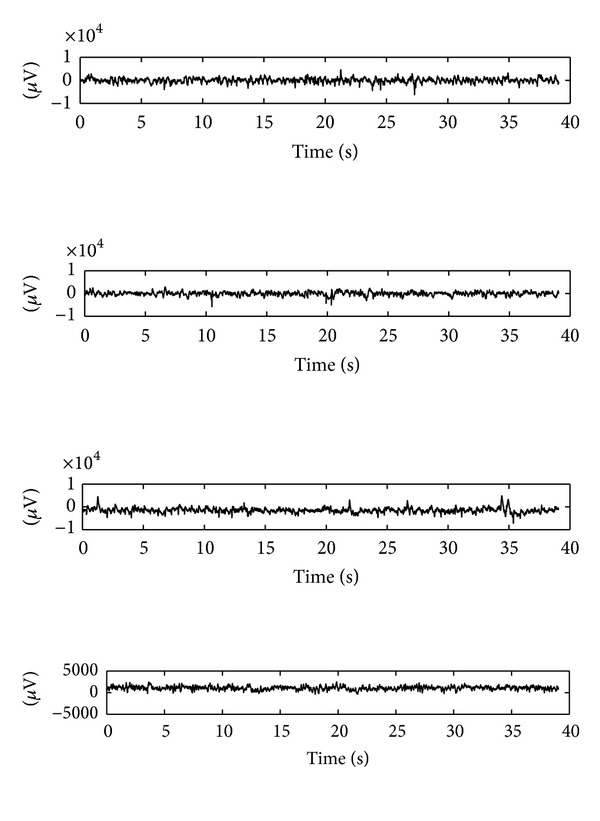
Examples of epileptic EEG segments from patients 1 and 3 of Freiburg dataset. From top to bottom: interictal EEG of patient 1, interictal EEG of patient 3, ictal EEG of patient 1, and ictal EEG of patient 3.

**Figure 4:**
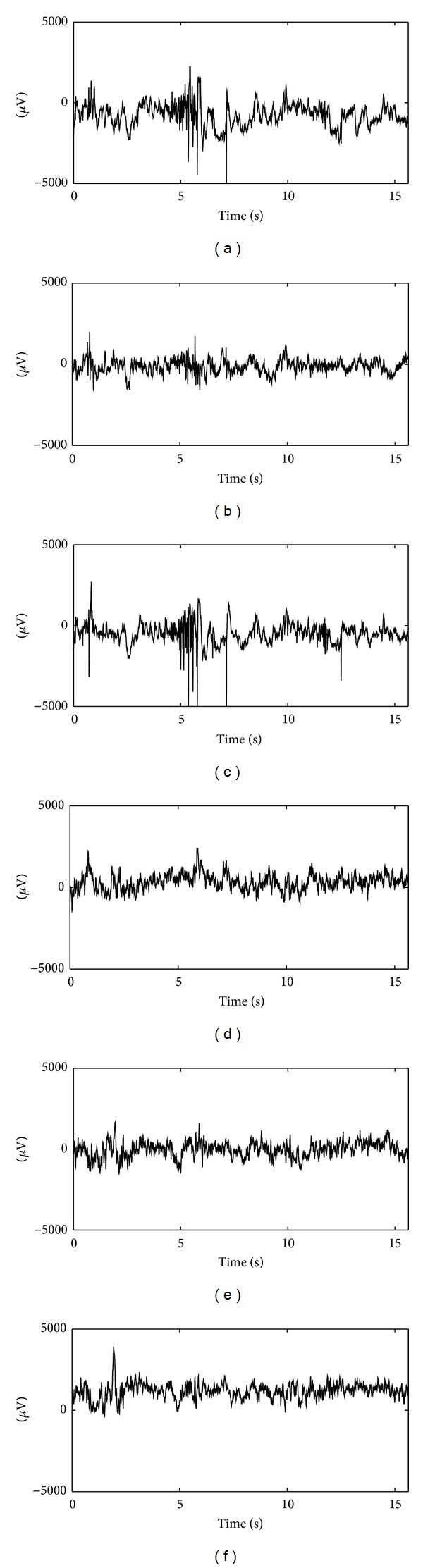
Examples of 6-channel epileptic EEG segments from patient 3 of Freiburg dataset. From top to bottom and left to right, they are channels 1–6, respectively.

The second EEG dataset (denoted by data set number 2) used here is a subset of EEG database available online from Seizure Prediction in Freiburg, Germany [15]. They are invasive EEG recordings of 21 patients suffering from medically intractable focal epilepsy. The dataset consists of 6 channels. Each interictal signal of those patients was sampled with a 256 Hz sampling rate. These observations are stored in data files with each being one hour in length. There are only 7 ictal signals for each patient and each signal is also one hour in length including stages before seizure, seizure, and after seizure. In the present work, only the EEGs from patients 1 and 3 are used due to large size of each data file. Examples of signal segment of patients 1 and 3 are displayed in Figure 3. From Figure 3 one can see that the signal differences are small between interictal EEGs and ictal EEGs and discrimination based on signal energy only is not a good choice. Examples of 6-channel epileptic EEG segments from patient 3 of Freiburg dataset are shown in Figure 4.

### 2.2. Dynamic PCA with Nonoverlapping Moving Windows

The dependence of measurements suggests that additional time-dependent variables should be introduced to long-term data analysis. In order to achieve the extraction of additional time-dependent variables, dynamic principal component analysis (DPCA) [5], which is often called singular spectrum analysis (SSA) in the time series analysis literature [16], is often used. In the method of DPCA or SSA, the variables being analyzed by PCA are the lagged versions of time series. More specifically, suppose that we have a collection of observations, {*y*(1), *y*(2),…, *y*(*N*)⊆ℝ} from a signal *y*(*n*) for 1 ≤ *n* ≤ *N*, where *N* is the number of observations. Theoritically speaking, the DPCA method assumes that the underlying signal is stationary and the input data matrix **Y** is organized as follows:

(1) Y=[y(n−l+1),y(n−l+2),…,y(n)],

for *n* = *l*, *l* + 1,…, *N*, where *l* is the time lag. The way that input matrix is being organized suggests that a signal is treated as a set of repeated overlapping windows. When *l* is chosen (often it is problem dependent), the number of variable of the underlying stochastic process is increased from 1 to *l*. Due to the need of dimension reduction, PCA is then applied to the covariance matrix of the data matrix **Y** to remove the insignificant singular values or the components that explain minor data variation. The idea of DPCA or SSA is to treat **Y** as a random vector and use PCA as a dimension reduction method.

Form matrix (1) one can see that the row data is highly dependent on each other. For example, when *n* = *l* and *l* + 1, the first row and the second row are *R*_1_ = [*y*(1), *y*(2),…, *y*(*l*)] and *R*_2_ = [*y*(2), *y*(3),…, *y*(*l* + 1)], respectively. Obviously, the row vectors in matrix (1) are highly autocorrelated. Moreover, since we deal with a long-term signal, *N* is typically large. The total row number of (1) is equal to *N* − 1 from the result of using DPCA with overlapping moving windows. This implies that there exists a very high redundancy in the input matrix so that the computation of finding principal components is less efficient.

In order to potentially improve the performance of DPCA, we propose a method of applying a non-overlapping moving window technique. Using the non-overlapping moving window technique, the data matrix constructed from the *N* observations of a signal *y*(*n*), denoted by **D** ^*y*^, is organized as follows:

(2) $$\begin{array}{c} D^{y}=\left(\begin{array}{cccc} y\left(1\right) & y\left(2\right) & \cdot \cdot \cdot & y\left(l\right)\\ y\left(1+l\right) & y\left(2+l\right) & \cdot \cdot \cdot & y\left(2l\right)\\ \vdots & \vdots & \vdots & \vdots \\ y\left(ml-l+1\right) & y\left(ml-l+2\right) & \cdot \cdot \cdot & y\left(ml\right) \end{array}\right), \end{array}$$

where *m* is the total number of the moving windows of *y*(*n*), *l* is the length of each moving window and *N* = *ml*. Each row of **D** ^*y*^ corresponds to a non-overlapping moving window. The observations of lagged variables within the window are time dependent and less autocorrelated when *l* is a larger value than the first significant time lag. Therefore, the dependencies among the rows of **D** ^*y*^ are less than the ones when over-lapping moving windows are used. This will reduce the effect of cross-correlation of moving windows in PCA so that the inferential performance of using PCA in an event detection problem may be improved. This is because it is a theoretical requirement to have uncorrelated observations in principal component analysis.

In the training step, suppose that there are *g* groups of signals and only *r* signals for each group. Since we deal with long-term signals and often *r* is small, a random allocation of signal is unrealistic. Instead, a random allocation is applied to the data segments. The data matrix constructed from these *r* signals becomes **D** = [**D** ^*y*_1_^^*⊤*^, **D** ^*y*_2_^^*⊤*^,…, **D** ^*y*_*gr*_^^*⊤*^]^*⊤*^, with the size *m* *gr* × *l*. Thus, each **D** ^*y*_*i*_^^*⊤*^, for *i* = 1,…, *r*, does not necessarily consist of the consecutive data windows so that the cross-correlation among windows is reduced. After organizing these signals into the data matrix **D**, PCA is then applied to map the matrix **D** into a new feature space. Due to the dimension reduction property of PCA, the number of extended variables from the time domain may be reduced. In PCA, the principal component score matrix **L** and the principal component loading matrix **V** = (*V*_1_,…, *V*_*l*_) are obtained by decomposing the *m* *gr* × *l* observation data matrix **D**, into **D** = **L** **V**. Sparse variable approximation via PCA is then obtained by approximating **D** by using a linear combination of first few components; that is $D\approx \hat{L}\hat{V}$, where $\hat{L}$ and $\hat{V}$ are low rank matrix and $\hat{V}$ consists of only first few PCs.

### 2.3. Detection Scheme Based on First Few Principal Components

After the PCs are extracted, each window of the test signal is then mapped onto the PC feature space to obtain the signal feature at each PC coordinate. If the test data is separable in the low-dimensional feature subspace, the extracted features are often clustered so that a simple classifier such as one nearest neighbor (1-NN) is able to classify them into the corresponding groups.

To perform PC extraction of a given test signal *s*(*n*) with a length N* = m*l, we organize the test data *s*(*n*) into a column vector, denoted by *Y* ^*s*^. We first partition *Y* ^*s*^ into m* windows each of length *l*; that is, *Y* ^*s*^ = [*Y* ^*s*^(1), *Y* ^*s*^(2),…, *Y* ^*s*^(m*)]^*⊤*^, where *Y* ^*s*^(*w*) = [*y*_1_^*s*^(*w*), *y*_2_^*s*^(*w*),…, *y*_*l*_^*s*^(*w*)] is the *w*th window of length *l* of *Y* ^*s*^, for each *w* = 1,2,…, m*. The objective of the PC extraction is to project each non-overlapping moving window of the test signal *Y* ^*s*^(*w*) onto the eigenvectors *V*_*v*_, for 1 ≤ *v* ≤ *l*, where *V*_*v*_ are obtained from eigenvalues decomposition of covariance matrix of the training data. For instance, the first principal component of *Y* ^*s*^(*w*) in the new feature space is the projection of *Y* ^*s*^(*w*) onto vector *V*_1_, denoted by ${\hat{y}}_{1}^{s}(w)$, the second principal component of *Y* ^*s*^(*w*) is the projection onto *V*_2_, denoted by ${\hat{y}}_{2}^{s}(w)$, and so on. These projections are the principal component scores in the PC feature space. However, due to the dimension reduction property of PCA most of the data variation is explained by the first few PCs. Therefore, one may extract only the important features of new observations using the first few projections. In this case, the number of the retained PC dimensions becomes l*, where l* ≪ *l*. We call this method a first few PCs (FFPC) sparse approximation method. When signals are highly correlated or stationary, the major data variations of the moving windows are explained by the first few PCs and the rest of the PCs are mainly corresponding to noises and could be dropped.

### 2.4. Detection Scheme Based on First Few PCs and Energy Measure

The DPCA with non-overlapping moving windows is an approach of analyzing the spectral structure of these moving windows. The underlying assumption for an optimal result is that these moving windows share a common finite-dimensional distribution. When signals are long-term observational, they are often nonstationary; for example, the expected signal energy of these moving windows may be varying. Therefore, a feature vector that consists of only the first few principal components may not lead to a successful classification. This is because the extracted features are similarity measures between the observed data and each principal component coordinate, which only capture the correlation structure of *l* random variables of the moving window. Often a large value of window size increases the chance of capturing important signal characteristics, but the percentage of data variation explained by these first few PCs becomes small as many of these *l* random variables of the window are less correlated. Therefore, retaining only a few PCs for classification may cause insufficient dimensions of discriminative features. In order to improve the separability of features used, a possible solution is to construct the feature vector containing the first few PCs, for example, first two PCs, ${\hat{y}}_{1}^{s}(w)$ and ${\hat{y}}_{2}^{s}(w)$, of the *w*th window plus the energy measures of the *w*th window in PC space, which is given as

(3) $$\begin{array}{c} E^{l}\left(w\right)=\sum_{v=1}^{l}{\hat{y}}_{v}^{s}{\left(w\right)}^{2}, \end{array}$$

where *w* = 1,2,…, m* is the index of the non-overlapping moving window of the test signal. Often the calculation of *E* ^*l*^(*w*) is not necessary up to *l*. For the data we consider, a value that is equal to half of *l* has been sufficient for providing discriminative energy measure. This approach extends the feature vector consisting of the first few PCs by additional one feature that measures the signal amplitudes. The classification performance that makes use of both the dependencies structure and the energy measures of moving windows may be potentially improved. We refer to this method as the first few PCs and energy measure (PCPEM) method.

### 2.5. Classification Methods

There are many classification methods such as *k*-nearest neighbors (*k*-NN) and the linear discriminant analysis (LDA) available for classifying the extracted features. Since our focus is on the extraction of low-dimensional feature vector, we only consider a simple classifier such as one nearest neighbor classifier. The one nearest neighbor classification method is a special case of *k*-NN. It is parameter-free and simply assigns the test object to the class of its nearest neighbor. We do not consider the simple classification methods such as LDA as this type of method involves another layer of feature transformation so that the significance of our proposed methods may become unclear.

## 3. Results

The methods are applied to dataset number 1 for the purpose of epilepsy diagnosis and epileptic seizure detection, two important event detection problems in epilepsy study. The long-term monitoring is studied using dataset number 2 as described in Section 2.1. In order to improve interpretability of signal features, we focus only on a low-dimensional feature vector, that is, a three-dimensional feature vector, as an input of data classification, to facilitate the real-world application as many monitoring systems would require a display of extracted feature in order to visually access the differences among them. We do not report the results of using the number of feature vector dimension less than three, because for most of the classification problems we deal with, the obtained classification accuracies are lower than the ones of using the three-dimensional feature vector. We also find that, for both EEG datasets, the increase of the feature vector dimension from three does not improve the classification performance; they are even worse for some cases. The results we report here are optimal by considering both the classification accuracy and the feature vector dimensions.

### 3.1. Epilepsy Diagnosis and Epileptic Seizure Detection

For each classification problem, that is, epilepsy diagnosis and seizure detection, the DPCA method partitions each EEG signal of Sets A, B, C, D, and E into a set of segments using a predefined window size *l*. The 10-fold cross-validation scheme is used to estimate the average classification accuracy, that is, the proportion of all signal types that are correctly classified. We compare the performance of data classification based on the FFPC method and the PCPEM method coupling with 1-NN classifier. From the scatter plots of the three features extracted shown in Figures 6(a) and 6(b) one can see that both the FFPC method and the PCPEM method may perform similarly in the diagnosis of epilepsy. However, the scatter plots of the three features extracted shown in Figures 6(c) and 6(d) suggest that classification based on the features from the PCPEM method may lead to a higher classification accuracy than those from the FFPC method. Replacing the third PC by the signal energy measure greatly improves the separability of the features used. This is due to the fact that the ictal signals have much higher signal energy than nonictal signals.  Table 1 reports the classification results for both epilepsy diagnosis and epileptic seizure detection problems under different values of *l*. The average accuracy has been reported for using both method in the form of mean ± standard deviation. In both applications, the PCPEM method outperforms the FFPC method in terms of both classification accuracy and the robustness of the approach. We also observe that with the increase of the window size, both of the detection methods reach their highest classification accuracy. The optimal results are obtained when the window length is *l* = 512, for both detection methods and both of the detection problems. This may also suggest that the methods are able to detect an epileptic seizure at a time range equal to the length *l*.

On the other hand, Table 3 reports the comparison of classification accuracy between our method and other methods proposed in the literature for the seizure detection problem. Most of the existing methods were applied for classification of normal type of signals (i.e., Set A) and the ictal type of signals (i.e., Set E), but we also consider the classification problem which combines all nonictal type signals (i.e., Sets A, B, C, D) as one group and ictal type (i.e., E) as another group, in order to reflect the problem of large signal variability in a real clinical application. For the classification problems considered in this work, our method has reached 100% accuracy. Our approach gives highly promising classification results using only the simple classifier, unlike other methods that often involve some complicated classification methods or learning systems. These facts may facilitate real-time monitoring applications in biomedicine.

### 3.2. Epilepsy Monitoring

The EEG data used for illustration of application to epilepsy diagnosis and seizure detection are short-term signals, which are extracted from a long-term EEG recording. In this case, the long-term signal variability caused by different stages of brain activities has been reduced, but it is possible to remove the information about different stages of brain activities or spatial location of epileptic EEG due to the well separation of obtained features. This is why we consider the classification of signals from ictal and nonictal periods in the precedent study of long-term monitoring. The mixing of normal EEG and abnormal EEG from preictal period introduces additional intersignal variability, which makes classification problem more complex. A successful classification of such problem will demonstrate the high potential for the proposed methods being used in the long-term monitoring. In this work the long-term time variability effect on the proposed techniques for seizure detection problem is also investigated. For this purpose, we use EEG from patient 1 and patient 3. As the classification problem in this study is based on a single-channel EEG, the long-term signal variability may be due to the lengthy observation time or may be because of different spatial location of EEG, as we allow the input of EEG for classification to be from different channels.

We choose *l* = 1280 as the selected window size for further study as this window size leads to the best result among all cases considered. This can be seen in Table 2, which reports the average classification under different values of *l*. For the effect of long-term signal variability due to different channels, we randomly select the desired number of one hour long EEG from all six channels of both interictal and ictal signals. The results with respect to different number of channels used and different method applied are reported in Table 4. The EEGs corresponding to different channels are randomly sampled from the dataset of each patient and each of them is a one hour long signal. The average accuracy for 10 trials has been reported for using both methods in the form of mean ± standard deviation. From this result, we observe that, the FFPC method performs better than the PCPEM method and it is less affected by the increase of number of channels used and the combination of EEG from different channels. From Table 4, one can see that the best performance is achieved when only two randomly selected channels are used. Also the FFPC method performs more robustly (i.e., less affected by subjects and the choice of channels; see the average in Table 4 for each method) than the PCPEM method. Another possible effect on long-term signal variability is the lengthy observation time. In order to investigate this, we focus on EEG of a fixed channel (i.e., channel 1) of each patient and randomly select the desired number of signals (each is one hour long). The results with the different length of observation time and different methods applied for feature extraction are summarized in Table 5. The results shown in Table 5 suggest that the FFPC method outperforms the PCPEM method as it performs more robustly as the observation time is increased and less affected by the intersubject differences.

However, the experimental results shown in Tables 1 and 2 suggest that the PCPEM method performs better on the dataset number 1 from the University of Bonn. This apart from the difference in classification accuracy in these two classification problems is due to the fact that each one hour long ictal signal from Freiburg data contains more than 50 min preictal data. But Set E contains only the seizure activity. The preictal signals are close to interictal signals in terms of signal amplitudes, but their dependencies structure within the signal may be different. The successful classification from interictal and ictal signals by using the FFPC method with the first three PCs indicates that signal dependencies structure of Freiburg data had more discrimination power than their signal energy measure. This apart from the difference may also suggest that the FFPC method is a better choice for long-term monitoring with off-line classification of signals and the PCPEM method is more suitable for long-term monitoring with real-time processing on short-period signals.

## 4. Discussion

In Section 2.2, the moving window of signal *y*(*n*) is treated as a discrete time-dependent function. This requires a set of less cross-correlated samples as PCA is affected primarily by the dependencies among the moving windows [17]. The direct use of DPCA is not appropriate as each row of (1) is highly correlated because the (*j* + 1)th row is obtained by shifting the *j*th row to the left by one time step, where *j* = 1,2,…, *N* − *l* + 1. As a result, the correlation among the row data in (1) may seriously affect the performance of estimating the eigenvalues and eigenvectors of the covariance matrix [17]. When *l* is small, the results may be particularly affected for multiscale signals because the extension of the number of variable from 1 to *l* may not be sufficient to capture the dynamical behaviors of signals. When *l* is large, often **Y** is a large scale matrix so that PCA of **Y** becomes computationally intensive. For example, for *l* = 512 and *N* = 4096, the size of data matrix **Y** is 3583 × 512; therefore, the expense on computing covariance matrix and decomposing it is dramatically increased. For the same *l* and *N*, the size of data matrix **D** ^*y*^ becomes 8 × 512, which is much easier to deal with. This proposed non-overlapping moving window technique is particularly useful for the PC extraction of a collection of long-term signals. Application of the DPCA approach allows extraction of additional variables in order to capture the complex structure of signals. This approach simultaneously extracts signal features from a set of windows obtained from multiple signals. From the computational point of view, it is more effective when compared with other signal approximation methods such as the matching pursuit and wavelet decomposition that deal with a single signal at a time.

**Figure 5:**
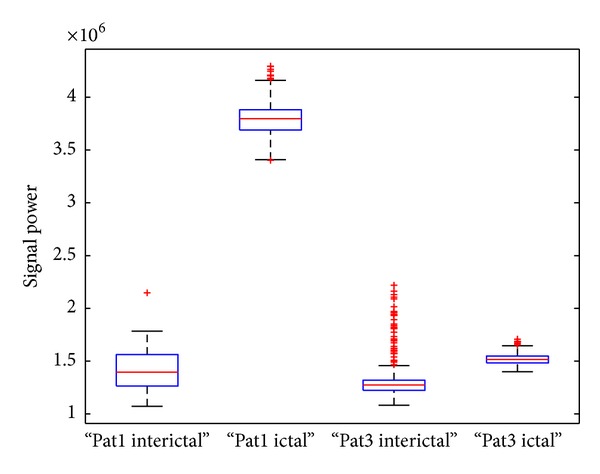
Box plots of average power of signal segments for patients 1 and 3. One hour long signal is used and *l* = 1280 is taken.

Since we do not classify signals based on all the PCs extracted, which are high-dimensional, our method improves the interpretability of the features as well as the potential improvement of the classification accuracy. In epilepsy seizure detection, the energy of ictal signals is much higher than both normal, seizure-free signals (e.g., see Figures 2 and 5). The inclusion of signal energy in the feature vector enables a separation of extracted features when signal energies are different from classes. The strength of this method is that it enables capturing data characteristics in terms of both the data variation and the signal energy measure, in the feature subspace.

This study explores the capability of applying DPCA based sparse variable approximation techniques coupled with a simple classifier in event detection problems from EEG. The DPCA method with non-overlapping moving window technique is applied to both short-term and long-term EEGs for seizure detection problem. The non-overlapping moving window technique helps reduce the correlations of windows in conventional DPCA. The proposed detection methods are highly promising in applications of epilepsy diagnosis and epileptic seizure detection.

**Figure 6:**
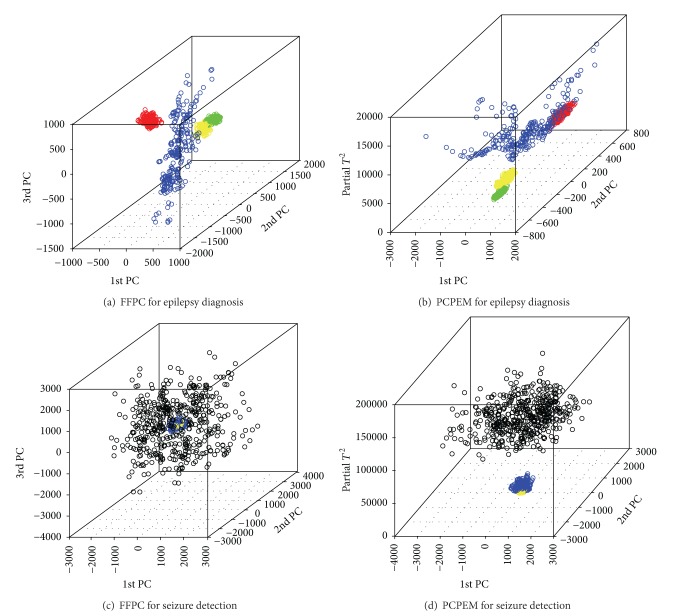
Three-dimensional features scatter plot obtained from the DPCA method for the test signals in Sets A, B, C, D and E for the FFPC method and for PCPEM method in both epilepsy diagnosis (using signals from Sets A, B, C and D) and seizure detection (using signals from Sets C, D and E). The scatter plots in green, red, yellow, blue and black colors stands for signals in Sets A, B, C and D and E, respectively.

As several characteristic EEG patterns are associated with well-defined epilepsy syndromes, it would be more clinically significant to classify EEG into more classes according to its corresponding epilepsy syndromes, which is important for selection of therapy and assessment of prognosis of the epilepsy. The results shown in Figure 6 demonstrate the possibility of classifying normal signals of different stages, seizure-free epileptic signals with different spatial location, and seizure type of signals into their respective group. Unlike the conventional epileptic seizure detection methods, the presented method can be expanded easily to accommodate multiclass classification of various IEDs without the significant loss of classification accuracy as PCA based method enables a group cluster in terms of different levels of data variation. Therefore, different stages of brain activities will form different clusters that facilitate the classification process.

## Figures and Tables

**Table 1:** The average classification accuracy (i.e., the proportion of all signal types correctly detected) using methods of FFPC and PCPEM with different values of l for the epileptic seizure detection and diagnosis problems using the EEGs dataset from the University of Bonn.

Seizure detection	l = 64	l = 128	l = 256	l = 512
FFPC	0.973 ± 0.003	0.982 ± 0.003	0.991 ± 0.001	0.994 ± 0.001
PCPEM	0.999 ± 0.001	1.000 ± 0.000	1.000 ± 0.000	1.000 ± 0.000

Epilepsy diagnosis	l = 64	l = 128	l = 256	l = 512

FFPC	0.699 ± 0.003	0.779 ± 0.036	0.959 ± 0.007	0.995 ± 0.009
PCPEM	0.732 ± 0.006	0.806 ± 0.003	0.978 ± 0.030	1.000 ± 0.000

**Table 2:** The classification accuracy (i.e., the proportion of all signal types correctly detected) using methods of FFPC and PCPEM with different values of l for the epileptic seizure detection problem using one hour long EEGs from Freiburg dataset.

Seizure detection	l = 512	l = 768	l = 1024	l = 1280
FFPC				
Patient 1	0.998 ± 0.001	1.000 ± 0.000	1.000 ± 0.000	1.000 ± 0.000
Patient 3	0.994 ± 0.017	1.000 ± 0.000	0.996 ± 0.009	1.000 ± 0.000
PCPEM				
Patient 1	0.986 ± 0.022	0.963 ± 0.043	0.983 ± 0.023	0.980 ± 0.026
Patient 3	0.987 ± 0.022	0.992 ± 0.008	0.980 ± 0.020	0.987 ± 0.019

**Table 3:** A comparison of classification accuracy obtained by various methods for epileptic seizure detection problem using EEGs dataset from the University of Bonn.

Papers	Method (feature extraction + classification method)	Problems	Accuracy
Nigam and Graupe [6]	Nonlinear preprocessing filter + diagnostic neural network	A–E	97.2%
Srinivasan et al. [7]	Time-frequency domain features + recurrent neural network	A–E	99.6%
Kannathal et al. [8]	Entropy measures + adaptive neurofuzzy inference system	A–E	92.22%
Polat and G$\ddot{\text{u}}$neş [9]	Fast Fourier transform + decision tree	A–E	98.72%
Subasi [10]	Discrete wavelet transform + mixture of expert model	A–E	95%
Tzallas et al. [11]	Time-frequency analysis + artificial neural network	A–E	100%
Guo et al. [12]	Multiwavelet transform and entropy + MLPNN	A–E	99.85%
This work	DPCA with PCPEM + 1-NN,	A–E	100%
Kim and Rosen [13]	AR model + PCA	B-C-E	96.6%

Tzallas et al. [11]	Time-frequency analysis + artificial neural network	A, B, C, D-E	97.73%
Guo et al. [12]	Multiwavelet transform and entropy + MLPNN	A, B, C, D-E	98.27%
This work	DPCA with PCPEM + 1-NN	A, B, C, D-E	100%

**Table 4:** The classification accuracy (i.e., the proportion of all signal types correctly detected) using methods of FFPC and PCPEM with window size l = 1280 for the epileptic seizure detection problem using EEGs from Freiburg dataset. The channels used for classification are randomly selected from those 6 channels.

Methods	2 Channels	3 Channels	4 Channels	5 Channels	6 Channels
FFPC					
Patient 1	0.999 ± 0.001	0.997 ± 0.010	1.000 ± 0.000	1.000 ± 0.000	1.000 ± 0.000
Patient 3	0.999 ± 0.003	0.997 ± 0.005	0.988 ± 0.007	0.990 ± 0.003	0.991 ± 0.003
Average	0.999 ± 0.002	0.997 ± 0.007	0.994 ± 0.003	0.995 ± 0.001	0.995 ± 0.001

PCPEM					
Patient 1	0.970 ± 0.031	0.957 ± 0.032	0.958 ± 0.027	0.974 ± 0.015	0.984 ± 0.002
Patient 3	0.950 ± 0.022	0.895 ± 0.043	0.899 ± 0.023	0.902 ± 0.018	0.892 ± 0.005
Average	0.996 ± 0.026	0.926 ± 0.037	0.929 ± 0.025	0.938 ± 0.016	0.938 ± 0.003

**Table 5:** The average classification accuracy (i.e., the proportion of all signal types correctly detected) using methods of FFPC and PCPEM with window size l = 1280 for the epileptic seizure detection problem using different length of EEGs from Freiburg dataset, randomly selected from patient 1, patient 3, and both (mixtures of EEGs from both patients).

Methods	r = 2 hours	r = 3 hours	r = 4 hours	r = 5 hours
FFPC				
Patient 1	1.000 ± 0.000	0.991 ± 0.013	0.954 ± 0.036	0.944 ± 0.031
Patient 3	0.996 ± 0.009	0.992 ± 0.014	0.984 ± 0.027	0.981 ± 0.026
Both	0.999 ± 0.003	0.981 ± 0.034	0.986 ± 0.024	0.973 ± 0.026
Average	0.998 ± 0.004	0.988 ± 0.020	0.975 ± 0.029	0.966 ± 0.028

PCPEM				
Patient 1	0.983 ± 0.023	0.960 ± 0.043	0.968 ± 0.032	0.897 ± 0.038
Patient 3	0.980 ± 0.020	0.977 ± 0.036	0.955 ± 0.048	0.934 ± 0.055
Both	0.979 ± 0.050	0.942 ± 0.053	0.931 ± 0.079	0.932 ± 0.054
Average	0.980 ± 0.031	0.960 ± 0.044	0.951 ± 0.053	0.921 ± 0.049

## References

[1] World Health Organization (2001). *Epilepsy: Aetiogy [Sic], Epidemiology and Prognosis*.

[2] Fisher RS, Van Emde Boas W, Blume W (2005). Epileptic seizures and epilepsy: definitions proposed by the International League Against Epilepsy (ILAE) and the International Bureau for Epilepsy (IBE). *Epilepsia*.

[3] Noachtar S, Rémi J (2009). The role of EEG in epilepsy: a critical review. *Epilepsy and Behavior*.

[4] Khan YU, Gotman J (2003). Wavelet based automatic seizure detection in intracerebral electroencephalogram. *Clinical Neurophysiology*.

[5] Ku W, Storer RH, Georgakis C (1995). Disturbance detection and isolation by dynamic principal component analysis. *Chemometrics and Intelligent Laboratory Systems*.

[6] Nigam VP, Graupe D (2004). A neural-network-based detection of epilepsy. *Neurological Research*.

[7] Srinivasan V, Eswaran C, Sriraam AN (2005). Artificial neural network based epileptic detection using time-domain and frequency-domain features. *Journal of Medical Systems*.

[8] Kannathal N, Choo ML, Acharya UR, Sadasivan PK (2005). Entropies for detection of epilepsy in EEG. *Computer Methods and Programs in Biomedicine*.

[9] Polat K, Güneş S (2007). Classification of epileptiform EEG using a hybrid system based on decision tree classifier and fast Fourier transform. *Applied Mathematics and Computation*.

[10] Subasi A (2007). EEG signal classification using wavelet feature extraction and a mixture of expert model. *Expert Systems with Applications*.

[11] Tzallas AT, Tsipouras MG, Fotiadis DI (2007). Automatic seizure detection based on time-frequency analysis and artificial neural networks. *Computational Intelligence and Neuroscience*.

[12] Guo L, Rivero D, Pazos A (2010). Epileptic seizure detection using multiwavelet transform based approximate entropy and artificial neural networks. *Journal of Neuroscience Methods*.

[13] Kim H, Rosen J Epileptic seizure detection—an AR model based algorithm for implantable device.

[14] Andrzejak RG, Lehnertz K, Mormann F, Rieke C, David P, Elger CE (2001). Indications of nonlinear deterministic and finite-dimensional structures in time series of brain electrical activity: dependence on recording region and brain state. *Physical Review E*.

[15] https://epilepsy.uni-freiburg.de/freiburg-seizure-prediction-project/eeg-database.

[16] Golyandina NE, Nekrutin VV, Zhigljavsky AA (2001). *Analysis of Time Series Structure. SSA and Related Techniques*.

[17] Lansangan JRG, Barrios EB (2009). Principal components analysis of nonstationary time series data. *Statistics and Computing*.

